# From the Understanding of Maternal Molecules and Mechanisms to Predicting Embryonic Development

**DOI:** 10.1002/rmb2.70026

**Published:** 2026-02-10

**Authors:** Yubao Wei, Akihiro Inoue, Kei Miyamoto

**Affiliations:** ^1^ Laboratory of Animal Reproductive Physiology, Faculty of Agriculture, Kyushu University Fukuoka Japan

**Keywords:** AI, assisted reproductive technology, developmental potential, maternal transcript, metabolism

## Abstract

**Background:**

Embryo quality is a critical determinant of successful outcomes in assisted reproductive technology (ART). Various molecular and cellular mechanisms in oocytes influence embryo quality, and their understanding can lead to the establishment of selection criteria for enhancing implantation rates.

**Methods:**

This review summarizes current knowledge on oocyte factors influencing embryo quality, including organelle function, chromosome segregation, maternal transcripts, metabolism, and gene regulation. We also discuss emerging techniques for assessing the fate of embryonic development, such as time‐lapse imaging, preimplantation genetic testing for aneuploidy (PGT‐A), and artificial intelligence (AI) or machine learning‐based prediction models.

**Main Findings:**

Embryo quality is often determined by maternal factors‐driven mechanisms that affect developmental potentials. Advanced technologies such as omics‐based profiling and AI‐driven analyses offer promising non‐invasive assessment tools for embryo quality.

**Conclusion:**

Integrating molecular diagnostics of maternal factors with traditional morphological evaluation can refine embryo selection, improving ART success rates. Future research should focus on minimally invasive biomarkers and personalized prediction models.

## 1 Introduction

Assisted reproductive technology (ART) has dramatically transformed the landscape of infertility treatment. Since the advent of in vitro fertilization (IVF), clinicians have gained unprecedented access to the earliest stages of human development. Despite improved culture systems and laboratory protocols, implantation and live birth rates remain relatively low, with only about 20%–40% of morphologically “ideal” embryos leading to successful pregnancies [1, 2]. A key limitation lies in the inability to accurately and non‐invasively assess embryo viability before transfer.

Traditional morphological grading systems, based on cleavage rate, blastomere symmetry, and degree of fragmentation, offer a subjective and sometimes inadequate measure of developmental potential [3]. In response, there has been a growing push toward data‐driven and biologically informative selection methods. Technologies such as time‐lapse imaging (TLI) allow dynamic assessment of cleavage events and morphokinetics [4], while preimplantation genetic testing for aneuploidy (PGT‐A) enhances selection by screening chromosomal integrity [5]. Furthermore, metabolic profiling of spent culture media, so‐called “metabolomic fingerprinting”, has been explored as a non‐invasive biomarker of embryo fitness [6, 7]. More recently, artificial intelligence (AI)‐based models trained on large TLI datasets have demonstrated promise in predicting implantation success with higher objectivity and reproducibility than conventional grading [8, 9]. However, a recent randomized controlled trial (RCT) reported that AI‐based embryo selection did not demonstrate noninferiority to conventional morphological assessment, highlighting the need for validation and standardization before adoption [10]. Nevertheless, these approaches could be further refined through multidisciplinary research. In particular, the biological mechanisms underlying the selection of high‐quality embryos should be clarified.

This review synthesizes current insights into the biological and technological factors that influence embryo viability. The embryo viability, namely developmental potentials of embryos, is shaped by a multifaceted network of intrinsic cellular mechanisms and extrinsic environmental conditions. While the fundamental role of oocyte quality in embryonic development is well established [11], recent studies have revealed maternal factors that affect the developmental potential of embryos [12, 13]. Maternal effect genes and their transcripts stored during oogenesis critically determine embryonic development. By governing mitochondrial activity, spindle integrity, and mRNA turnover, maternal factors establish the developmental potential of the fertilized oocyte.

These elements coordinate to support key transitions during preimplantation development, including zygotic genome activation (ZGA). We here explore the maternal factors that serve as molecular underpinnings of early embryogenesis, particularly the roles of genomic stability, mitochondrial function, epigenetic regulation, and metabolic adaptability, and assess modern innovations in embryo selection. Most mechanistic insights into maternal factors discussed here originate from mouse models. Whenever available, corresponding evidence from human oocytes and embryos is also presented to highlight translational relevance. Ultimately, by improving the prediction of developmental potential, ART outcomes can be optimized, reducing physical, emotional, and financial burdens on patients.

## 2 Maternal Factors Related to the Developmental Potential of Embryos

Mammalian oocytes not only provide half of the DNA to the next generation, but also contain abundant maternal factors critical for meiosis, fertilization, and early embryonic development [14, 15]. Maternal RNAs and proteins stored during oogenesis drive development through the early cleavage stages until the maternal‐to‐zygotic transition (MZT). Recent work in mice has shown that the Endoplasmic Reticulum (ER) stress sensor IRE1α cleaves maternal mRNAs after fertilization, and the loss of RNase activity of IRE1α causes developmental arrest during the 1–2 cell stage due to failures of maternal mRNA clearance [14], suggesting that maternal mRNA degradation is key for development.

The oocyte‐specific or early‐embryo‐specific features of organelles are also regarded as critical determinants for subsequent development. For example, early mammalian embryos exhibit a functionally incomplete spindle assembly checkpoint (SAC), making the first divisions prone to chromosome mis‐segregation [16, 17, 18]. It should be noted that mouse oocytes lack canonical centrosomes, whereas human embryos inherit sperm‐derived centrosomes, leading to species‐specific differences in spindle organization. Embryos can sometimes sequester mis‐segregated chromosomes into micronuclei and eliminate them via cellular fragmentation, a phenomenon documented in primate embryos [19], but many aneuploid human blastocysts nevertheless fail to implant or lead to miscarriage.

The quality of the intracellular milieu, from organelle function and maternal RNA content to chromosomal integrity, critically governs embryo developmental potential in mammals [15, 19]. In this section, we introduce oocyte‐specific molecular and cellular features and discuss how these can potentially affect early embryonic development (Table 1).

**TABLE 1:** Maternal factors related to the developmental potential of embryos.

Maternal factors	Roles	Human evidences	Non‐human evidences
Mitochondria	Energy‐producing and maternal heredity of mtDNA	mtDNA contents could serve as a biomarker of embryo viability [20]. High mtDNA contents show lower developmental potential [21]	Signaling pathways and metabolic intermediates of Mitochondria maintain the energy metabolism, cell lineage allocation, and blastocyst formation [22]
Cortical granules (CGs)	Cortical reaction to prevent polyspermy	The cortical reaction, hardening the zona pellucida and blocking additional sperm entry [23]	Cortical reaction blocks additional sperm entry and participates in embryonic cleavage and development [24]
Cytoplasmic lattice (CPL)	Storage and regulated usage of ooplasmic ribosomes and mRNAs	CPL proteins are critical for oocytes and embryo development; mutations of related genes lead to embryonic arrest [25]	CPL proteins encoded by maternal effect genes are critical for oocyte and embryo development [26, 27]
Smooth endoplasmic reticulum (SER)	Ca^2+^ storage and release site, source of Ca^2+^ oscillations	Abnormal SER in oocytes has been linked to low‐quality embryo and lower clinical pregnancy rates [28, 29, 30]	SER could facilitate efficient local Ca^2+^ release and uptake [31]
Spindle assembly checkpoint (SAC)	Ensure fidelity of chromosomal segregation	Mitotic spindle disruption during mitosis activates the SAC in day‐3 and later embryos [32]	The SAC signaling could be maintained by AURKB during meiosis I [17]
Maternal transcripts	Supporting the synthesis of proteins in preimplantation embryos	Maternal mRNAs are remodeled dramatically at first cleavage stage [33]. Maternal mRNA clearance is pivotal during maternal‐to‐zygotic transition [34]	Maternal RNAs deposited in mature oocytes for maternal‐to‐zygotic transition [35]
Subcortical maternal complex (SCMC)	Multi‐protein assembly beneath the oocyte cortex, a functional module for maternal‐to‐zygotic transition	The maternal‐effect proteins, NLRP7 and KHDC3L, are localized at the cortical region in human oocytes and mutations of SCMC proteins would cause embryonic arrest [36, 37, 38]	The SCMC modulates the transition from oocyte to embryo by regulating a series of cellular processes [36, 39]

### 2.1 Oocyte Organelles

Oocytes mobilize a multitude of maternal factors after fertilization to support early embryonic development, and among these, the structure, distribution, and function of intracellular organelles play a critical role in determining the developmental potential of the fertilized egg. In recent years, there has been growing interest in the structural characteristics of these organelles and their functional implications. This section provides an overview of how cellular structures within the oocyte influence embryonic developmental competence, focusing on four key components: mitochondria, cortical granules (CGs), cytoplasmic lattices (CPL), and smooth endoplasmic reticulum (SER), based on the latest findings.

#### 2.1.1 Mitochondria

Mitochondria, the energy‐producing organelles of the cell, are almost exclusively derived from oocytes and play a pivotal role in early embryo development. Researchers have found that the amount of mitochondrial DNA (mtDNA) within an embryo can be an indicator of its viability. Two independent studies in human IVF embryos reported a striking pattern: embryos with abnormally high mtDNA contents tend to have lower developmental potential and implantation success [21]. In fact, a threshold appears to exist in human embryos, above a certain mtDNA copy number, embryos rarely implant, whereas those that successfully implant have significantly lower mtDNA levels [20]. This counterintuitive finding (more mtDNA *correlating with* less viability) observed in humans led to the concept that “A little goes a long way” for mtDNA in embryos. In clinical research, a “MitoScore” grading system was proposed to quantify this effect in human embryos, observing that embryos with mtDNA scores above the cutoff failed to implant [21]. However, clinical applicability of the MitoScore remains limited, and it is still considered an emerging and developing assessment method.

Collectively, these human studies suggested that mtDNA contents could serve as a biomarker of embryo viability, helping to identify abnormal blastocysts that are unlikely to result in a pregnancy [20]. The underlying rationale is consistent with the “quiet embryo” hypothesis, which proposes that healthier embryos function at a lower metabolic rate and therefore require fewer mitochondria. In contrast, embryos experiencing stress tend to increase mitochondrial activity and mtDNA copy number, a pattern that is associated with reduced developmental viability. While high mtDNA levels are regarded as a warning sign, they reflect cellular stress or inefficiency rather than cause it.

The mitochondrial transfer approach has been suggested to boost mitochondrial functions in oocytes with compromised quality. Historically, cytoplasmic (ooplasmic) transfer has been developed to improve the oocyte quality by supplementing cellular components from a healthy oocyte [40]. The heterologous ooplasmic transfer has resulted in pregnancies in humans but has raised concerns due to persistent donor‐derived mtDNA (heteroplasmy) [40, 41, 42]. Consequently, a more refined and ethically acceptable technique—autologous mitochondrial transfer—was developed. In this approach, mitochondria derived from the patient's own cells, typically from ovarian somatic cells such as granulosa cells, and in some protocols from putative oogonial stem cells (OSCs) in ovarian tissue (the existence of functional OSCs in adult human ovaries remains controversial [43]), are injected into the oocyte at the time of intracytoplasmic sperm injection (ICSI). A recent study applied this approach using OSCs in patients with recurrent IVF failure and observed remarkable improvements in embryo quality [44]. After mitochondrial supplementation, the treated oocytes produced embryos with significantly high quality and blastocyst formation rates, and ultimately 13 healthy babies were born to the previously unsuccessful patients [44, 45]. Importantly, the safety of the procedure was supported by genetic evidence showing that the mtDNA sequences of the babies were almost identical to those of their mothers. This finding indicates that there was no harmful carryover or mutations introduced by the added mitochondria [44, 46]. These results demonstrate that mitochondrial transfer can be an effective option to enhance embryo quality and IVF outcomes in certain cases.

Maintaining mitochondria in a healthy state is clearly essential for embryo development. One factor that can compromise mitochondrial integrity in oocytes and embryos is oxidative stress, which refers to an imbalance between the reactive oxygen species (ROS) and the capacity of antioxidant defenses. Excess ROS directly damage mitochondrial DNA and internal structures, leading to a vicious cycle of mitochondrial dysfunction: damaged mitochondria produce energy less efficiently and may generate even more ROS [47]. Empirical evidence links such oxidative damage to poorer embryo performance. For instance, mammalian embryos that experience high oxidative stress show delayed blastocyst development and reduced quality, likely because of accumulated cellular damages and the cellular failure to clear out the dysfunctional mitochondria [48]. IVF‐derived mouse blastocysts exhibit elevated ROS, reduced mitochondrial respiration, and increased glycolysis, which correlate with lower developmental competence compared with in vivo embryos [49].

#### 2.1.2 Cortical Granules (CGs)

CGs are Golgi‐derived secretory vesicles that migrate to and accumulate in the subcortical region of mature oocytes [24]. Upon fertilization, CGs rapidly fuse with the oocyte plasma membrane and release enzymes that trigger the zona pellucida reaction, referred to as the cortical reaction, hardening the zona and establishing a block to additional sperm entry [23, 24]. In human oocytes, CGs form a suboolemmal layer whose correct positioning and exocytosis are essential for monospermic fertilization. Recent ultrastructural studies have shown that CG mislocalization or premature release is observed after cytoskeletal defects or reproductive aging [50, 51]. Moreover, laboratory studies indicate that cryopreservation can perturb CG integrity in human oocytes, with implications for the polyspermy block and embryo quality [52]. In aged or in vitro matured oocytes from animal models such as mouse and pig, CGs' positioning and exocytotic competence are disrupted, leading to abnormal CG release, lowered fertilization rates, and increased developmental arrest [53, 54]. Whether similar cortical granule defects occur in aging human oocytes remains to be determined. For example, ovastacin is a cortical granule–localized metalloprotease that is first identified in mouse that cleaves ZP2 and must be released to modify the zona pellucida. If ovastacin is not properly released, post‐fertilization zona hardening is impaired [55]. Likewise, transglutaminase 2 (TGM2) contributes to the prevention of polyspermy by crosslinking zona pellucida proteins based on pig model studies [56]. Inhibition of TGM2 has been shown to increase the incidence of polyspermy, whereas treatment with recombinant TGM2 promotes zona hardening and reduces polyspermy in porcine oocytes. The extent to which TGM2 plays a similar anti‐polyspermy role in human oocytes remains to be confirmed. Membrane fusion of cortical granules is mediated by Soluble N‐ethylmaleimide‐sensitive factor Attachment protein Receptor (SNARE) complexes in the oocyte, a mechanism conserved across mammalian species. A study demonstrated that SNARE proteins VAMP1 and VAMP3 are required for cortical granule exocytosis in mouse oocytes, whereas VAMP2 is not [57].

Post‐ovulatory aging leads to reduced autophagic activity, mis‐localization of cortical granules and ovastacin, and poor fertilization outcomes. Recent studies in porcine oocytes have linked autophagy to the quality of cortical granules. Indeed, enhancing autophagy with spermidine has been shown to restore proper distribution of cortical granules and ovastacin [54], thereby improving oocyte quality. Thus, quantitative or qualitative defects in cortical granules have direct effects on embryo competence from the moment of fertilization, and monitoring CG maturation or cortical accumulation is being explored as a predictor of oocyte developmental potential [58].

#### 2.1.3 Cytoplasmic Lattice (CPL)

Mammalian oocytes contain a unique fibrillar structure called the CPL, which is absent from somatic cells [59]. The CPL is enriched in oocyte‐specific maternal proteins and RNAs and has long been hypothesized to function as a storage depot for the maternal stock of ribosomes and transcripts needed after fertilization. Indeed, genetic studies in mice have shown that maternal effect genes expressed in oocytes, such as PADI6, NLRP5 (MATER), TLE6, KHDC3L (FILIA), and OOEP (FLOPED), encode core CPL components or their associated factors [60, 61, 62, 63, 64].

In mice, PADI6 protein localizes to the CPL and is required for its formation: PADI6‐null oocytes lack visible CPLs and females are infertile with embryos arresting at the two‐cell stage [59]. Similarly, loss of NLRP5 prevents normal CPL assembly, and Ooep‐null oocytes completely lack CPL fibril [26]. TLE6 is another CPL/subcortical maternal complex (SCMC) component essential for embryogenesis: null mutations in mouse Tle6 cause early cleavage arrest, and biallelic TLE6 variants in women lead to recurrent preimplantation embryonic arrest [25, 65]. Collectively, these findings from both mouse models and human patients establish that core CPL proteins encoded by maternal‐effect genes are critical for oocyte developmental competence and progression past the first mitotic divisions [26, 27].

The CPL appears to coordinate the storage and regulated use of ooplasm ribosomes and mRNAs. Ultrastructural and biochemical evidence indicates that a majority of oocyte ribosomes are sequestered in the CPL rather than engaged in translation during oogenesis [59]. In wild‐type mouse oocytes, PADI6 helps “pack” ribosomal components into the CPL. In PADI6‐deficient oocytes, ribosomal proteins and ribosomal RNAs become mislocalized, and many maternal ribosomes fail to associate with the insoluble lattice complex. PADI6‐null two‐cell mouse embryos show reduced de novo protein synthesis and dysregulated translation of specific maternal mRNAs such as abnormally high spindlin production [59]. These data from the mouse model imply that the CPL normally organizes the embryonic translational machinery so that stored ribosomes and selected transcripts are mobilized properly during early cleavage. The CPL also contains RNA‐binding regulators: for instance, the oocyte mRNA‐binding protein MSY2 co‐localizes with the lattice, and in PADI6‐null mouse oocytes MSY2 is severely mislocalized [66]. Thus, the CPL and its associated factors provide a scaffold for controlled storage and timely translation or turnover of maternal mRNAs before ZGA, a function demonstrated in mice that is likely conserved across mammalian species.

Deficiency of CPL components impairs the MZT. Mouse models deficient in PADI6, NLRP5, OOEP, TLE6 or related maternal genes all show developmental arrest around the 2‐cell stage [59]. In these mutants, the first cleavage is delayed or abnormal, and embryos fail to activate zygotic transcription. In PADI6‐null 2‐cell embryos, nuclear RNA Polymerase II, especially its phosphorylated active form, is significantly reduced, indicating defective ZGA [66]. Similarly, human IVF patients with biallelic mutations in TLE6 or NLRP5 have normal fertilization, but their embryos arrest before the blastocyst stage [25]. These clinical observations reinforce that intact CPL machinery is essential for early development.

CPL‐associated proteins are emerging as biomarkers for developmental ability, especially in human IVF. Several studies in humans now link variants or expression of SCMC/CPL genes (like PADI6 and TLE6) to embryo competence. For example, maternally inherited PADI6 loss‐of‐function mutations not only cause abnormal epigenetic reprogramming but also early embryonic arrest, demonstrated in mouse models, highlighting its importance for normal preimplantation development [67]. In clinical cohorts, women with recurrent IVF failure often harbor pathogenic variants in CPL components, and embryos from these patients fail to progress preimplantation development presumably due to the abnormal embryonic gene regulation [25, 68]. These findings suggest that assaying CPL‐related maternal factors could help predict human oocyte/embryo viability in ART.

In summary, the oocyte CPL is a specialized maternal architecture composed of factors like PADI6, NLRP5, OOEP, TLE6, and KHDC3L that orchestrate ribosomal and mRNA storage and translational control. Its proper assembly is critical for early embryogenesis, demonstrated in mice and likely true in humans. Loss of CPL function disrupts maternal mRNA regulation, diminishes protein synthesis and ZGA, and results in developmental arrest [59, 62]. The presence and integrity of CPL components are thus integral to fertility, and their disruption is likely to underlie many cases of early embryonic loss across species.

#### 2.1.4 Smooth Endoplasmic Reticulum (SER)

In mammalian oocytes, the smooth endoplasmic reticulum (SER) in oocytes serves as a major Ca^2+^ storage and release site, and functions as the source of Ca^2+^ oscillations observed at fertilization [31]. These Ca^2+^ signals are crucial for triggering oocyte activation, spindle assembly, cell cycle resumption, and the initiation of early embryonic development. In mature mammalian oocytes, the SER has been observed to form clusters with surrounding mitochondria, facilitating efficient local Ca^2+^ release and uptake [31, 69].

Abnormal SER structures in human oocytes have been reported to affect oocyte quality and subsequent embryo development. For example, in one clinical study, human oocytes with SER clusters had significantly lower fertilization rate (73.9% vs. 86.2%), lower good‐quality blastocyst rate (26.7% vs. 44.1%), and slower development compared with unaffected oocytes [28]. Likewise, the retrospective cohort study shows that the presence of SER aggregates (SERa) in human oocytes is associated with decreased high‐quality day‐3 embryo rates [29]. One meta‐analysis in humans reported that the risk of major birth defects in children from SERa+ oocytes was significantly increased compared with SERa– oocytes [70]. Conversely, other large cohort studies in IVF patients found no significant differences in birth defect rates or live birth rates between SERa+ and SERa– groups [30]. Interestingly, the presence of SER aggregates has not been found to significantly affect the chromosomal aneuploidy rate of embryos [29].

The SER physically contacts mitochondria via its associated membranes, enabling localized Ca^2+^ transfer and metabolic control. This localized regulation of ATP production and translational activity is thought to help establish the cytoplasmic environment necessary for fertilization and embryogenesis [69]. SER abnormalities in human oocytes should thus be viewed not merely as a morphological oddity but as potential indicators of oocyte functional competence. Accordingly, in IVF practice, evaluating SER status should involve not only noting its presence but also assessing features such as the frequency of occurrence, spatial distribution, structural morphology, and co‐localization with mitochondria for oocyte quality assessment. Such detailed observation of SER may allow each patient to select a high‐quality oocyte for further treatment or cancel the subsequent embryo transfer due to the presence of SERa [71].

Thus, the various organelles within the oocyte function not merely as structural components but as central determinants of embryonic developmental fate through their multifaceted physiological roles, including energy production, translational regulation, calcium mobilization, and mechanisms preventing polyspermy. The structural and functional maturity of these organelles can serve as an indicator of oocyte quality as well as a decisive factor governing the developmental competence of the resulting embryo. Therefore, comprehensive evaluations of these organelles hold significant importance from both reproductive medicine and developmental biology perspectives.

### 2.2 Chromosome Segregation

Meiotic chromosome segregation in oocytes is a fundamental biological process that ensures the accurate transmission of genetic material to the next generation. The fidelity of chromosomal segregation is ensured by the spindle assembly checkpoint (SAC), which prevents progression through meiosis until all chromosomes are correctly attached to microtubules [17]. The SAC in oocytes is notably more permissive than in somatic cells (demonstrated in animal models), allowing progression even when some chromosomes are not properly aligned. This reduced stringency becomes exacerbated with maternal aging, leading to a sharp rise in segregation errors and aneuploidy, which are major contributors to infertility, miscarriages, and congenital abnormalities in older women [17, 18].

A key regulator of SAC activity is Aurora B kinase (AURKB), which functions at inner centromeres to monitor microtubule attachment fidelity and promotes SAC signaling, thereby preventing premature cell cycle progression [72, 73]. In aged mouse oocytes, loss or downregulation of AURKB results in reduced SAC protein levels, weakened SAC signaling, and an increased frequency of mis‐segregation during meiosis I [17]. This decline in AURKB function with age directly contributes to the breakdown of SAC surveillance and the accumulation of aneuploid oocytes in **mice**; a similar phenomenon is likely in human oocyte aging, although the extent remains under investigation [17, 74]. Aurora A kinase (AURKA), a closely related kinase, plays a distinct but equally critical role in mouse oocytes by regulating acentriolar microtubule organizing centers (aMTOCs) dynamics, thereby enabling proper bipolar spindle assembly in the absence of centrosomes. In mouse oocytes, AURKA regulates the distribution of aMTOCs and the localization of TACC3, a protein required for spindle building [75]. Recent studies in mouse oocytes further show that AURKA phosphorylates the kinetochore protein HEC1 (NDC80) at serine 69 during meiosis I, enhancing both chromosome alignment and SAC activation [76]. These findings underscore the dual role of AURKA in spindle assembly and SAC satisfaction through regulation of kinetochore signaling.

In addition to checkpoint surveillance, physical cohesion between sister chromatids is crucial for proper segregation. This cohesion is mediated by the meiosis‐specific cohesin complex, comprising several proteins including SMC1β, SMC3, STAG3, and the kleisin subunit REC8 [77]. Since oocytes arrest in prophase I for extended periods (decades in humans, months in mice), cohesin proteins deteriorate over time, leading to age‐dependent weakening of chromatid cohesion. Aged oocytes in both mice and humans exhibit reduced centromeric REC8 and SGO2 localization, leading to premature sister chromatid separation and mis‐segregation [78, 79]. In human oocytes, the protection of centromeric cohesion is partly mediated by Shugoshin 2, whose loss from centromeres in aged oocytes contributes to increased chromatid dissociation and aneuploidy [79]. Not all chromosomes are equally susceptible to mis‐segregation. Acrocentric chromosomes, such as human chromosomes 13, 14, 15, 21, and 22, have structural features that make them more prone to breakage. These chromosomes have centromeres located near the short arms and tend to possess fewer cohesin complexes than metacentric counterparts, shown in porcine eggs [80]. Consequently, they are more sensitive to age‐related cohesion loss and mis‐attachment to spindle fibers, making them particularly prone to mis‐segregation. For example, chromosome 21 mis‐segregation is a leading cause of Down syndrome [80].

Altogether, the fidelity of chromosome segregation in oocytes is regulated by an integrated network involving SAC stringency mediated by AURKB, proper spindle assembly through AURKA, the integrity of cohesin complexes such as REC8 and SGO2, and the intrinsic structure of chromosomes. Disruption in any of these components, such as the simultaneous decline in SAC activity and cohesin function during aging, can lead to severe mis‐segregation events. Advanced maternal age in humans is often associated with cumulative impairments across these mechanisms, resulting in higher rates of aneuploid oocytes, reduced embryo quality, implantation failure, and an increased risk of chromosomal disorders [17].

### 2.3 Maternal Transcripts

Maternal RNAs deposited in mature oocytes are deployed under tight post‐transcriptional control from fertilization through ZGA, forming the molecular basis for developmental competence. In early embryos, cytoplasmic polyadenylation element‐binding protein 1 (CPEB1) and DAZL regulate translation of mouse maternal mRNAs, thereby supporting the synthesis of proteins required for development [35]. The BTG4/CCR4–NOT deadenylase complex may remove unneeded maternal transcripts via a maternally encoded decay pathway (M‐decay) before ZGA as observed in human oocytes [34]. For example, in mouse oocytes, BTG4 recruits CCR4–NOT to actively translating maternal mRNAs and accelerates their deadenylation, licensing their timely degradation and proper MZT progression [81, 82, 83].

Structural components associated with the CPLs, including PADI6 and the RNA‐binding proteins ZAR1 and ZAR2, contribute to the concentration and stabilization of maternal mRNAs in the oocyte cytoplasm. These components help organize a scaffolded environment that supports regulated translation. PADI6 is a key component of both the SCMC and the CPLs. In mice lacking PADI6, CPLs do not form, and embryonic development arrests at the two‐cell stage [67]. This finding suggests that the integrity of CPLs is crucial for maintaining maternal mRNA stability, facilitating normal ZGA [67]. Similarly, in mice, ZAR1 and ZAR2 bind to and stabilize subsets of maternal transcripts [84]. In mouse oocytes lacking both ZAR1 and ZAR2, the levels of many maternal mRNAs, including the MZT licensing factor BTG4, are reduced. In addition, translation of these transcripts is impaired, resulting in defective MZT [84].

SCMC is a multi‐protein assembly beneath the oocyte cortex (originally characterized in mice and also present in human oocytes), composed of maternally derived factors such as TLE6, PADI6, and MATER [39, 62]. Each SCMC contributes to essential early developmental processes, including the organization of cytoplasmic architecture, proper cell division, and the establishment of polarity. Loss of any core SCMC factor in mice disrupts these processes and prevents progression beyond the initial zygotic cleavage stages. The SCMC is present in oocytes before ZGA and provides a cytoplasmic scaffold that also assists in the positioning and translational control of maternal mRNAs (a role defined in mouse models and likely conserved in humans). In particular, loss of PADI6 or other SCMC components disrupts CPL formation, leading to destabilization of maternal transcripts and delayed or failed ZGA, which is observed in mouse knockouts and mirrored by early embryonic arrest in patients with maternal‐effect gene mutations [36, 37, 67].

After ZGA, a zygotic decay pathway (“Z‐decay”) clears remaining maternal mRNAs. In mouse embryos, the maternal factor YAP1 and zygotic factor TEAD4 induce expression of terminal uridylyl transferases TUT4/7, which mediate uridylation at the 3′ ends of maternal mRNAs and trigger their degradation [85, 86]. Importantly, not all maternal transcripts are eliminated in the earliest divisions. Recent studies in mice have identified the “MARTRE” family of proteins as guardians of poly(A) tail length during the oocyte‐to‐embryo transition. MARTRE proteins bind directly to the CCR4‐NOT deadenylation complex and inhibit its activity, thereby protecting long poly(A) tails on maternal mRNAs and sustaining their translation [87]. In MARTRE null mouse embryos, actively translated maternal mRNAs acquire shorter poly(A) tails and exhibit reduced translation, leading to delayed 2‐cell progression and compromised preimplantation development. This reveals a novel mechanism in mice by which embryos fine‐tune the timing of MZT by preserving maternal mRNA translation until zygotic control is fully established.

SCMC components continue to support the embryo even after ZGA (observed in early development of both mouse and human embryos). TLE6, MATER, NLRP2, PADI6, and other SCMC proteins remain functionally important in the early embryo, contributing to the maintenance of chromosome/genome integrity, organelle distribution, and blastocyst formation [36, 62]; evidence primarily from mouse models, with some indications in humans [39]. The SCMC serves not only as a structural scaffold but also as a regulator of maternal mRNA localization and translation, ensuring the quality and developmental potential of early embryos [88].

### 2.4 Other Aspects

In addition to the roles of intracellular organelles, chromosome segregation mechanisms, and maternal transcripts, recent research highlights other determinants of oocyte and embryo developmental competence. In particular, the plasticity of the oocyte's epigenome, the functionality of autophagy, and the cytoplasmic homeostasis, including pH and redox balance, are critical. For instance, epigenetic marks established during oogenesis, particularly histone modifications and DNA methylation, are essential for proper ZGA and the expression of imprinted genes [89, 90]. Disruption of these marks, whether due to maternal aging or in vitro culture conditions, can increase the risk of abnormal embryonic development [91, 92, 93].

The maintenance of autophagy is also directly linked to developmental potential. In post‐ovulatory mouse oocytes, the VPS34 pathway normally functions to clear damaged organelles [94]. Oocyte‐specific deletion of VPS34 impairs both autophagy and mitophagy, resulting in the accumulation of defective mitochondria with elevated levels of ROS, reduced mitochondrial membrane potential, and a marked decrease in ATP production and protein synthesis in mammalian MII oocytes and their early embryos [95]. Consequently, such embryos fail to complete ZGA and arrest at the 2–4 cell stage in mice. Similarly, perturbations of the cytoplasmic redox environment affect mitochondrial function and translation: oxidative stress elevates ROS and induces mitochondrial dysfunction, suppressing ATP production and protein synthesis and correlating with reduced developmental competence [95, 96]. Metabolic support from cumulus cells is also essential for oocyte maturation and embryo development. Transzonal projections (TZPs) and gap junctions facilitate the transfer of nutrients from cumulus cells to the oocyte. In aging mouse ovaries, a reduction in the number of TZPs and the smoothing of microfilaments in the zona pellucida impair sperm binding, which leads to significantly lower fertilization rates and reduced embryo viability [97].

**FIGURE 1:**
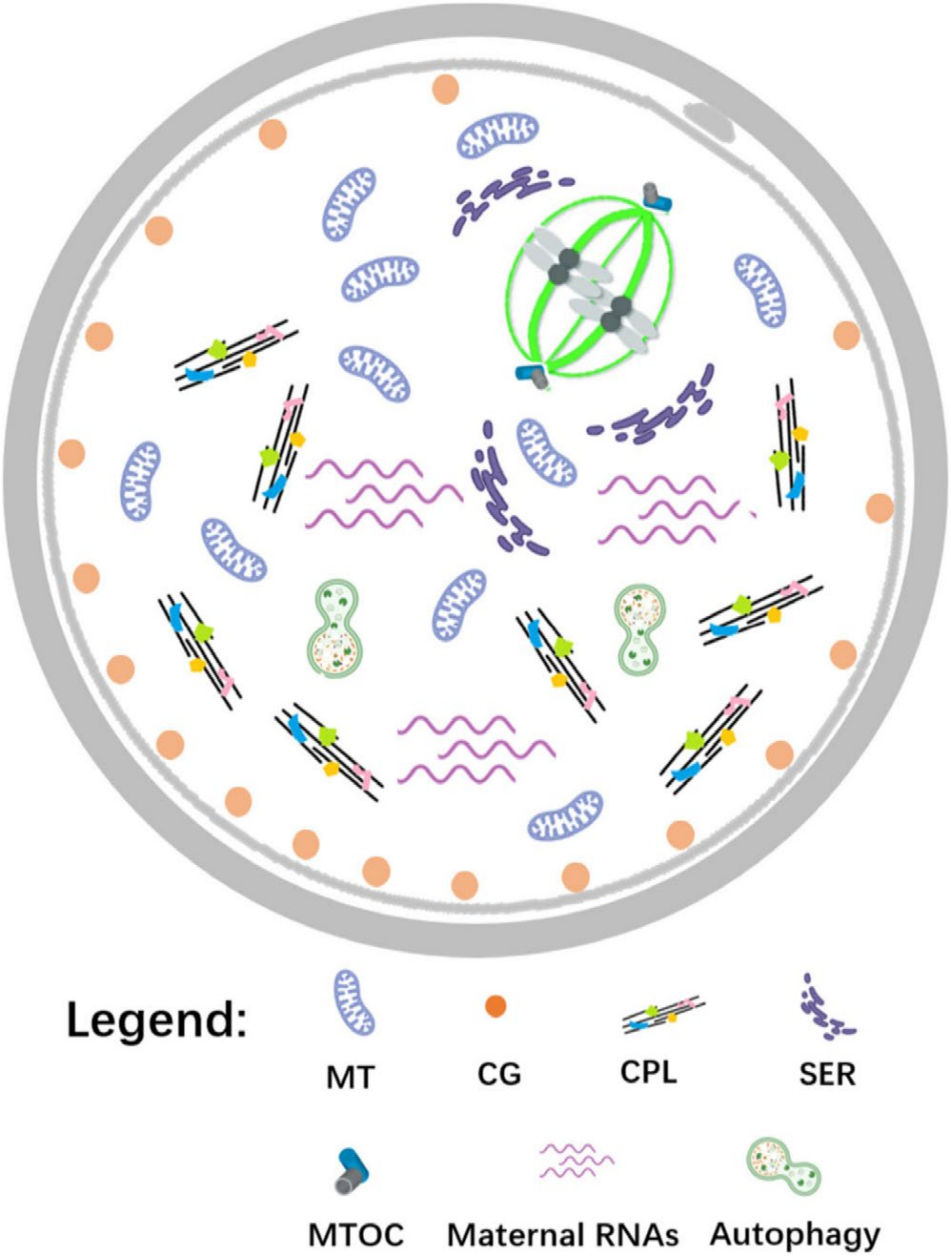
Multiple oocyte factors that can influence embryo developmental potential. These factors are categorized into oocyte organelles, chromosome segregation, maternal factors, etc. Abbreviations: MT: mitochondria; CG: cortical granule; CPL: cytoplasmic lattice; SER: smooth endoplasmic reticulum; MTOC: microtubule organizing center.

Although each of these factors may cause only subtle effects on its own, their combined disruptions ultimately determine oocyte quality. Therefore, comprehensive and multidimensional analyses that include organelle function, chromosome and spindle integrity, maternal transcript abundance, epigenetic regulation, autophagy, intracellular homeostasis, and cumulus–oocyte interactions are essential to reliably assess the developmental potential of an oocyte (Figure 1).

## 3 Assessment of the Developmental Potential of Embryos

The previous chapter summarized maternal factors relevant to the developmental potential of an oocyte after fertilization. Understanding the molecular mechanisms by which maternal factors regulate oocyte competence provides not only fundamental biological insight but also a foundation for developing more objective and predictive evaluation methods for embryonic development. As advances in ART increasingly rely on quantitative, data‐driven assessments, linking biological mechanisms to measurable phenotypes has become essential.

Maternal factors fundamentally influence the early cellular and molecular events that determine embryo morphology, kinetics, and genomic stability. These biological features are increasingly being captured by modern embryo assessment technologies. In TLI, variations in cleavage timing, blastomere symmetry, and compaction dynamics often reflect underlying differences in maternal mitochondrial activity, spindle organization, and mRNA clearance efficiency—core determinants of oocyte competence. PGT provides information on genetic normality/abnormality [98]; chromosomal abnormalities and mtDNA copy number variations can result from errors in maternal gene products that control meiotic division, mitochondrial replication, and DNA repair. AI‐based prediction models, trained on large‐scale morphokinetic or transcriptomic datasets, may therefore detect morphological or molecular signatures arising from these maternal factors. Integrating such a mechanistic understanding of maternal biology with advanced predictive tools could improve both the biological interpretability and clinical reliability of embryo selection systems.

Accurate assessment of the embryo developmental potential remains a major challenge in ART. Effective evaluation helps reduce recurrent miscarriage, optimize implantation rates, and improve live birth outcomes. This section summarizes four major approaches for assessing the developmental ability of human embryos: TLI, PGT‐A, machine learning–based prediction systems, and emerging minimally invasive or noninvasive techniques.

### 3.1 Time‐Lapse Imaging

TLI, or time‐lapse monitoring, utilizes incubators equipped with integrated microscopes to continuously capture images of preimplantation development without disturbing culture conditions or impairing developmental potential [99]. Unlike conventional microscopy, which requires embryos to be periodically removed from the incubator, TLI captures high‐resolution images at regular intervals (e.g., every 5–60 min) from fertilization through the blastocyst stage [100]. TLI allows detailed analysis of morphokinetic parameters, including the timing of pronuclear fading (tPNf), cleavage to the 2‐, 4‐, 8‐cell stages, morula compaction, and blastocyst formation [101].

Retrospective studies and systematic reviews have shown that early cleavage timings and synchronous cell divisions are associated with higher rates of pregnancy in humans [102]. A systematic review of 47 studies concluded that morphokinetic parameters derived from TLI are associated with embryo viability indicators, including blastulation, implantation, and live birth [102]. In general, a systematic review provides the highest level of evidence compared with other types of research, according to the evidence pyramid. Despite its objectivity and standardization, RCTs have not consistently demonstrated improvement in live birth outcomes compared with conventional morphology‐based selection [103]. Additionally, confounding factors such as maternal age, culture conditions, and uterine environment independently affect outcomes. TLI facilitates elective single‐embryo transfer (eSET) by identifying embryos with optimal kinetic profiles, but the overall quality of evidence remains low [103, 104]. In sum, while TLI shows potential, existing RCTs have not yet demonstrated consistent benefits, and further research is needed.

### 3.2 Preimplantation Genetic Testing for Aneuploidy

PGT‐A involves biopsy of trophectoderm cells (typically at day 5 blastocyst stage) to assess chromosomal copy number using next‐generation sequencing (NGS), array comparative genomic hybridization (aCGH), or single‐nucleotide polymorphism (SNP) arrays [105, 106]. Earlier PGT approaches based on fluorescence in situ hybridization (FISH) targeted a limited number of chromosomes and did not show clear clinical benefit. With the advent of comprehensive 24‐chromosome analysis, the use of PGT‐A increased substantially, from 14% of U.S. IVF cycles in 2014 to 44% in 2019 [107].

PGT‐A aims to reduce miscarriage risk by avoiding the transfer of aneuploid embryos. While early single‐center studies suggested higher live birth rates in specific subpopulations (e.g., advanced maternal age), recent multicenter RCTs have produced inconsistent results [107]. New directions, such as PGT for polygenic diseases (PGT‐P) using genome‐wide polygenic databases drawn from a few cells, to predict later‐life traits and disease risks [108]. But their scientific validity and ethical implications remain controversial [109].

Concerns over diagnostic accuracy persist, including false positives and mosaic classification errors [110]. Several reasons for PGT‐A yielding false negatives and mosaic results have been documented [111]. For example, the investigation of trophectoderm cells, which form the placenta, may not accurately reflect the embryo's ploidy [111]. The mosaic nature of euploid and aneuploid cells within an embryo [111] can increase the risk of false positives. Furthermore, it is noted that embryos selectively get rid of aneuploid cells [19, 111], and the detection of such aneuploid cells by PGT‐A does not necessarily mean the abnormal embryos. In addition to these concerns, there are technical limitations to accurately detecting copy number variations [112]. Notably, retrospective analyses have reported normal deliveries following transfer of embryos previously labeled as mosaic aneuploid by PGT‐A [111, 113]. In one retrospective analysis, embryos labeled aneuploid by PGT‐A later resulted in normal delivery [114]. The evidence level of retrospective analyses is moderate and lower than that of RCTs, and further comprehensive studies are needed. In sum, PGT‐A seems best applied selectively, for example, to women of advanced age, recurrent miscarriage, or known chromosomal rearrangements. Nonetheless, PGT‐A should be viewed as a screening rather than a definitive diagnostic tool. Genetic counseling and a clear understanding of its limitations are essential before clinical use.

### 3.3 Machine Learning–Based Prediction

In recent years, machine learning techniques have been increasingly applied to ART and embryology, particularly in human IVF, using embryo images and patient data to predict developmental outcomes and select the most viable embryo [115, 116]. For example, AI models trained on TLI can automate the ranking of human embryos by extracting features from microscopy images, aiming to identify those with the highest chances of successful pregnancy [117]. Deep learning methods present applications such as classifying mouse embryos according to developmental stages with approximately 88% accuracy [118]. In humans, a single blastocyst image can be used to estimate the chance of live birth, with a prediction score of 67% accuracy [119].

A substantial number of studies in human IVF aim to forecast clinical outcomes such as pregnancy or implantation [120]. These studies predominantly use TLI of preimplantation stage embryos as input data (mostly from human IVF cases). A convolutional neural network (CNN) is reported to be the most common approach for analyzing embryo images. CNN‐based models, including pretrained networks, as well as other deep learning architectures, have been trained to evaluate embryo morphology and to identify subtle morphokinetic patterns. Notably, models integrating multiple data types perform best. The fusion model significantly outperformed the image‐only or data‐only models, improving the prediction of clinical pregnancy and live birth [116]. This demonstrates that, *in human IVF* applications, AI can take advantage of both embryo morphology and patient factors such as age and stimulation protocols to enhance prediction outcomes.

In addition to CNN, other machine learning methods have also been explored. For example, in human IVF, tree‐based ensemble models such as LightGBM and XGBoost are used to predict the number of blastocysts produced per IVF cycle, based on patient characteristics and cycle‐related variables [121]. Here, cycle‐related variables refer to treatment‐specific factors within each IVF attempt, such as the ovarian stimulation protocol, dosage and duration of gonadotropins, number of oocytes retrieved, fertilization method (IVF or ICSI), and culture conditions [121]. These models are supposed to be superior to linear regression by capturing nonlinear interactions. Advanced imaging techniques have also been adopted. For instance, the EVATOM system combined quantitative phase imaging with machine learning to assess embryo health without the use of labels, achieving a very high accuracy in classifying mouse embryos as high‐quality or lower‐quality in a proof of concept study [122].

In summary, both human and mouse studies have demonstrated that a variety of machine learning approaches, including CNN for image classification, gradient‐boosting algorithms, and hybrid models, can provide predictions of developmental stages, embryo viability, and clinical outcomes [123]. These advances hold significant potential to enhance embryo selection and support patient counseling in the context of ART. A summary of the key machine learning techniques, target species, predicted outcomes, and practical applications from recent studies is provided in Table 2.

**TABLE 2:** AI tools to predict embryo viability and ART outcomes.

Prediction name	Learning method	Predictive contents	Learning content
Analysis of age‐related changes in the embryo developmental speed [124]	XGBoost (Gradient Boosting)	Maternal age classification (young vs. old)	Time‐lapse microscopy images
Prediction of blastocyst formation from Day3 embryos [125]	ResNet‐GRU (Deep Learning)	Blastocyst formation (developing to Day5)	Time‐lapse images from Day0 to 3
Pipeline for embryo viability classification from images and clinical data [126]	CNN (EfficientNet‐B0) + SimCLR + GAN (Transfer Learning)	Embryo viability (implantation potential)	Microscopy images + clinical features
Label‐free imaging‐based embryo assessment tool “EVATOM” [122]	CNN (EfficientNet‐B7) + feature model	Embryo health status (healthy/unhealthy)	Label‐free optical phase images (GLIM)
AI model for live birth prediction and Day5 embryo selection [127]	CNN (SGD Training)	Live birth after single embryo transfer	Time‐lapse images of Day5 blastocysts
Automatic early embryo detection and hierarchical classification [128]	Haar + CNN (Embryo detection & cell number classification)	Embryo stage classification by cell number	Time‐lapse images from Day2 to 3 embryos
Pregnancy prediction from nuclear morphology using LSTM combined with an Attention mechanism [129]	NVAN (Normalized Multi‐View Attention Network)	Live birth after transfer	Time‐series nuclear morphology data
Prediction of fetal heartbeat using time‐lapse video (IVY model) [8]	CNN (IVY; Attention Branch Network)	Clinical pregnancy (fetal heartbeat)	Time‐lapse video of Day5 blastocysts
Pregnancy prediction model integrating image and clinical data [119]	CNN (EfficientNet) + MLP integration	Clinical pregnancy (fetal heartbeat)	Day5 blastocyst images + patient clinical data
Blastocyst formation prediction from early morphology and clinical data [130]	Logistic Regression Model	Blastocyst formation (developing to Day5)	Early cleavage morphology + maternal age
Blastocyst count prediction from IVF cycle data [121]	LightGBM (Tree model)	The number of blastocysts	IVF/ICSI cycle clinical data
Embryo selection from single image with expert‐level performance [131]	CNN (ResNet)	Embryo selection (identifying high‐quality embryos)	Static Day5 blastocyst images
Blastocyst prediction using cytoplasmic movement speed [132]	k‐NN, LSTM, Ensemble NN	Blastocyst formation (development/arrest)	Cytoplasmic movement speed of early cleaving embryos via time‐lapse
Life Whisperer AI model for embryo viability and pregnancy prediction [133]	CNN (ResNet‐152)	Implantation success (viability)	Static Day5 blastocyst images
Implantation prediction using morphodynamic features [134]	Artificial Neural Network	Implantation prediction (single embryo transfer)	Time‐series features from the zygote to blastocyst stage

Abbreviations: ART, assisted reproductive technology; CNN, convolutional neural network; GAN, generative adversarial network; GLIM, gradient light interference microscopy; GRU, gated recurrent unit; ICSI, intracytoplasmic sperm injection; IVF, in vitro fertilization; IVY, Integrated Validation with Attention Branch Network (IVY model); k‐NN, k‐Nearest Neighbors; LightGBM, Light Gradient Boosting Machine; LSTM, long short‐term memory; MLP, multilayer perceptron; NN, neural network; NVAN, normalized multi‐view attention network; ResNet, residual neural network; SGD, stochastic gradient descent; SimCLR, Simple framework for Contrastive Learning of visual Representations; XGBoost, Extreme Gradient Boosting.

### 3.4 New Venues for Minimally Invasive Methods for Prediction

Recent research has focused on developing minimally or non‐invasive methods for assessing embryo viability, including analyses of cell‐free DNA, RNA, proteins, or metabolites in spent culture media [135]. Noninvasive PGT‐A using blastocoel fluid or spent medium shows potential but is limited by mosaicism and contamination, necessitating further validation.

Metabolomics and proteomics of spent culture media, using Raman or near‐infrared spectroscopy, have been explored as non‐invasive measures of embryo viability, though reproducibility across laboratories remains inconsistent [136, 137]. Proteomic approaches targeting secreted proteins such as haptoglobin or early embryo‐specific markers are still in the research phase. Advancements in imaging technologies, including 4D light‐sheet microscopy and label‐free polarization imaging, now permit high‐resolution evaluation without staining or biopsy [100].

Together, these technologies illustrate a paradigm shift toward more objective and data‐rich embryo assessment. TLI provides morphokinetic insight without disturbance but requires validation in RCTs. PGT‐A offers definitive genetic screening but must be used judiciously due to limitations in accuracy and ethical considerations. AI‐based models offer scalable and standardized embryo ranking systems but require transparency, consensus validation, and prospective trials. The progress in this field will depend on integrated multimodal approaches combining TLI, genomics, metabolomics, self‐supervised AI, and longitudinal patient outcomes. Development of transparent, interpretable predictive models aligned with robust RCT validation and regulatory guidelines is essential.

## 4 Future Directions

**FIGURE 2:**
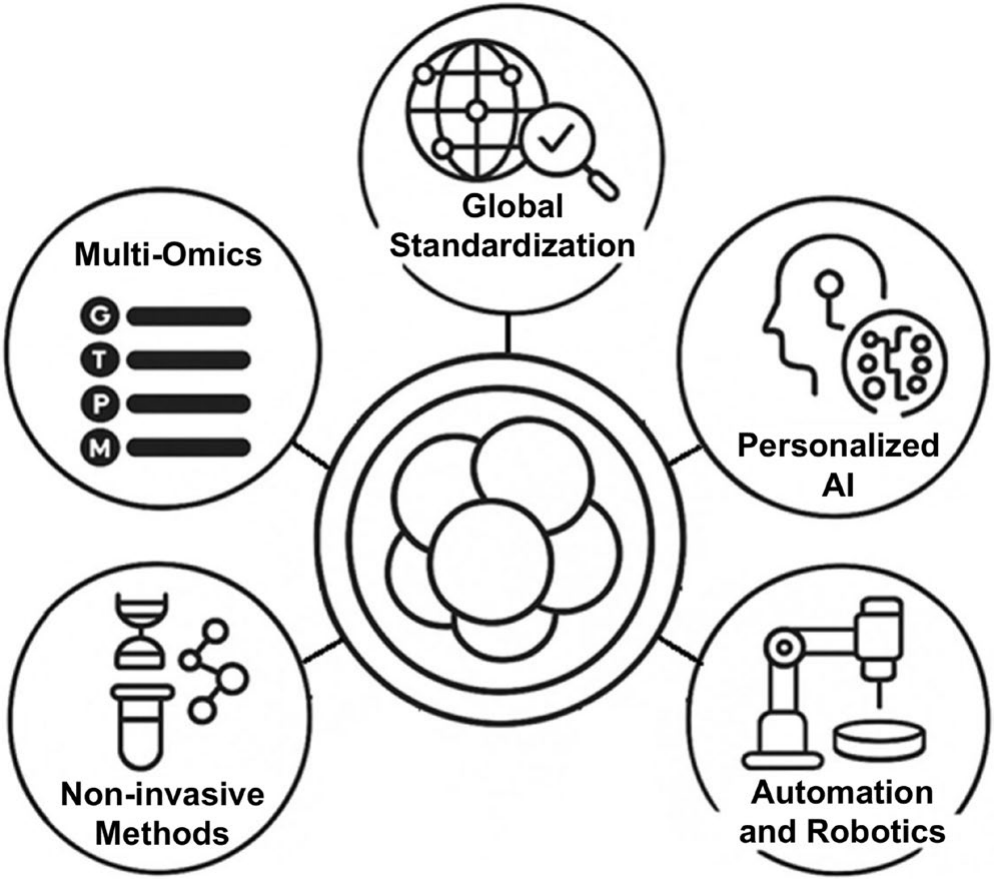
Future directions for embryo assessment and selection. The integration of multi‐omics technologies: Including genomics (G), transcriptomics (T), proteomics (P), and metabolomics (M). Noninvasive methods like cell‐free DNA analysis and metabolomic profiling of spent culture media provide alternatives to embryo biopsy. Advances in AI and machine learning enable personalized embryo selection using patient‐specific clinical data and time‐lapse imaging. Automation and robotics reduce variability in embryo handling and improve procedural consistency. Global standardization, through shared databases, FAIR data principles, and unified scoring systems, will enhance reproducibility and equity across ART centers.

Embryo assessment in ART is undergoing a profound transformation from subjective morphological evaluation toward objective, data‐driven, and integrated analytic technologies. Sharing anonymized databases from multicenter trials under FAIR (Findable, Accessible, Interoperable, Reusable) principles would facilitate the validation process and model reproducibility [138]. Center‐specific machine learning models trained on local data could outperform generalized models [139], but models trained on local data sets may not generalize across other fertility clinics. Universal scoring systems that combine multiple modalities into a single viability index are urgently needed. The ultimate goal remains consistent: maximizing live birth rates while minimizing risks, costs, and ethical concerns. As we approach a new era in ART, the most promising directions include multimodal integration, non‐invasive biomarkers, personalized AI, automation, and global standardization. Each area offers unique opportunities and challenges for next‐generation embryo selection and reproductive outcomes (Figure 2).

No single biomarker can fully capture embryo viability. Combining morphokinetic features with metabolic profiles increases prediction accuracy for implantation potential [140, 141, 142]. For multi‐omics integration to become routine, standardized collection protocols, data normalization, and bioinformatics pipelines are essential. For example, spent culture media must be consistently collected, processed, and stored to ensure reliable metabolomic analysis [143]. Harmonized gene references, imputation techniques, and machine learning models will improve reproducibility across centers.

Personalized embryo selection based on big data and AI represents a promising frontier. Patient‐specific factors such as age, infertility diagnosis, ovarian reserve, and endometrial receptivity should inform individualized prediction models. A major barrier to clinical adoption is the “black box” nature of AI. Efforts are underway to make model outputs interpretable by highlighting key image regions and temporal features [9]. Explainable AI, compliant with General Data Protection Regulation (GDPR), will improve clinician trust and regulatory acceptance.

Automation also holds transformative potential. Robotic‐assisted micromanipulation could standardize PGT‐A biopsy and embryo transfer. In 2023, the first babies conceived via robotic‐assisted ICSI were reported [144]. Pilot studies show that robotics delivers consistent handling quality and reduced procedure time, and minimizes human bias [145]. AI‐guided clinical decision support systems that generate embryo viability scores or recommended embryo handling steps could reduce practice variability [8, 146]. However, implementing automation will require substantial investment in hardware, software, and personnel training.

Regulatory and ethical oversight is fundamental for reproductive medicine. Strong regulatory frameworks, including informed consent, donor selection, and follow‐up surveillance, are critical to ensure patient safety and ethical responsibility. Harmonizing global ART standards—including training, scoring criteria, and reporting frameworks such as consolidated standards of reporting trials (CONSORT)—will reduce regional disparities in success rates [147]. International collaboration and tele‐mentoring can strengthen capacity in low‐ and middle‐income regions.

While the pace of innovation is promising in reproductive medicine, rigorous clinical trials, regulatory compliance, and economic evaluation remain essential. The ultimate goal is to enhance live birth rates for patients undergoing ART while minimizing the costs and procedural risks. Achieving this will require sustained interdisciplinary collaboration across embryology, genomics, bioengineering, and clinical medicine.

## Funding

This work was supported by Japan Society for the Promotion of Science (JP19H05751, JP20K2137), Japan Science and Technology Agency (JPMJFR233F), Takeda Science Foundation, Naito Foundation, and Japan Agency for Medical Research and Development (S1‐425008‐00).

## Conflicts of Interest

The authors declare no conflicts of interest.

## Data Availability

Data sharing not applicable to this article as no datasets were generated or analyzed during the current study.
